# Virtual Reality for Developing Patient-facing Communication Skills in a Medical Science Graduate Education Course: A Mixed-Methods Pre-Post Study

**DOI:** 10.1007/s40670-025-02604-4

**Published:** 2025-12-29

**Authors:** Kyla Gaeul Lee, Maryam Sorkhou, Nicole Harnett, Sobiga Vyravanathan, Theodore J. Brown, Evan Tannenbaum, Nairy Khodabakhshian

**Affiliations:** 1 Institute of Medical Science, University of Toronto, Temerty Faculty of Medicine, Toronto, C. David Naylor Building 6 Queen’s Park Crescent, Suite 119, M5S 3H2 Canada; 2 Department of Psychiatry, Sinai Health, Toronto, Canada; 3 Centre for Addiction and Mental Health, Institute for Mental Health and Policy Research, Toronto, Canada; 4 Princess Margaret Cancer Centre, Radiation Medicine Program, Toronto, Canada; 5 Department of Radiation Oncology, University of Toronto, Toronto, Canada; 6 Sinai Health, Lunenfeld-Tanenbaum Research Institute, Toronto, Canada; 7 Department of Obstetrics and Gynaecology, University of Toronto, Temerty Faculty of Medicine, Toronto, Canada; 8 Department of Obstetrics and Gynaecology, Sinai Health, Toronto, Canada; 9 Division of Cardiology, The Hospital for Sick Children, Toronto, Canada

**Keywords:** Virtual reality, Medical education, Communication, Patient-facing, Cultural safety

## Abstract

Communication skills are essential for patient-centered clinical research, yet traditional teaching methods offer limited opportunities for trainees to strengthen this competency. This study evaluates the impact of virtual reality (VR) modules on enhancing communication skills among graduate research trainees in medical science. A mixed-methods pre-post design was used to triangulate quantitative and qualitative data. Pre- and post-course scores for readiness and knowledge (Winter: *n* = 11, Fall: *n* = 29) were analyzed using a paired sample t-test. Qualitative data were collected during a class debriefing. Our quantitative findings revealed significant improvements in post-course scores for both knowledge (*p* < .001) and readiness for clinical integration (*p* < .05) compared to pre-course scores. Qualitatively, students described the modules as realistic, immersive, and engaging. However, they faced challenges in distinguishing their roles as researchers versus clinicians and in addressing cultural nuances during informed consent. VR-based learning improved students’ confidence and preparedness for real-world clinical research. Findings suggest the need for comprehensive education on informed consent and a stronger focus on ethical communication and culturally safe research practices. The modules also encouraged deep self-reflection, prompting students to confront their biases and their impact on participant inclusion.

**Supplementary Information:**

The online version contains supplementary material available at 10.1007/s40670-025-02604-4.

## Introduction

Graduate research programs in the medical sciences include a growing number of students engaged in clinical research, highlighting the need to prepare these trainees for the unique demands of healthcare environments. Graduate clinical researchers have responsibilities that are distinct from those of clinical practitioners. While practitioners focus on direct patient care, clinical researchers are responsible for designing and conducting studies that generate evidence to inform clinical practice. Their work requires obtaining informed consent, communicating study aims and procedures to participants, and interpreting findings for both scientific and lay audiences. These tasks demand a high level of clinical communication skills, as researchers must navigate ethical considerations, cultural contexts, and complex information to ensure that participants are adequately informed and engaged throughout the research process. Despite the centrality of these skills to research integrity and participant safety, they are often insufficiently developed through traditional graduate training approaches.

Many research-based graduate programs within medical science rely on inconsistent methods of building these skills, such as incidental observation of others in the clinical setting or classroom lectures, which can provide theoretical knowledge but may fall short in preparing students for the nuanced challenges of real-world healthcare environments [1, 2]. Further, incidental observations are often difficult to regulate and rarely the same between two students, resulting in potential inconsistencies in learning experiences [3]. There are also often administrative barriers that delay the learning process and also put additional strain on healthcare workers to train graduate students [4]. This shortfall highlights the need for innovative, experiential learning methods that can better equip students to handle the demands of clinical communication in diverse and dynamic healthcare settings while minimizing the impact on the system.

Virtual reality (VR) has emerged as a transformative technology in medical education, offering an interactive and immersive approach to addressing these training gaps. VR refers to a digital simulation technology that immerses users in a three-dimensional, interactive environment, replicating real-world situations [5]. By engaging learners in realistic clinical and research scenarios, VR allows students to practice essential communication skills in a controlled, low-risk setting. Structured and repeatable simulations also enable learners to practice delivering sensitive information, manage cultural nuances, and obtain informed consent with clarity and empathy, all without compromising patient safety. With this in mind, we developed a multiformat course for graduate students that combines lectures with interactive facilitated discussions, real-time group work, and several VR modules that mimic real-life scenarios that graduate students may encounter during their clinical experiences. This new course, ‘Clinical Research Skills’, is a 0.25-credit modular course designed for students to complete ideally in their first or second semester of graduate school, before beginning the more complex stages of their clinical research activities.

Although VR has been utilized in various medical education contexts, including surgical training and patient interactions, there is limited research specifically examining its effectiveness in enhancing communication skills within healthcare research settings. This study aims to address this gap by evaluating the impact of a comprehensive course that integrates VR technology to enhance clinical communication skills among graduate students in a medical science program. Using mixed methods approaches, our research triangulates quantitative findings with qualitative field notes to provide a more nuanced understanding of the course’s impact on students’ perceived communication abilities, confidence, and attitudes toward VR as a teaching tool. The findings aim to provide valuable insights into the potential of VR-enhanced courses in medical education, contributing to the development of more effective strategies for preparing students to navigate the complex communication demands in healthcare research and clinical practice environments.

## Methods

### Study Design

A mixed methods pre-post study design was used to evaluate the effectiveness of the MSC1121H Clinical Research Skills course in strengthening graduate students’ communication skills for patient-facing research. Research ethics approval was obtained from the University of Toronto Research Ethics Board (Protocol #: 00047058). The study examined first- and second-year graduate students who had completed the course over two terms. Quantitative measures captured changes in students’ knowledge and readiness for clinical integration. Qualitative data from class debrief sessions had explored students’ experiences in navigating the virtual reality modules, their perceptions of ethical and cultural challenges, and reflections on how these scenarios shaped their understanding of patient communication in clinical research settings. These two approaches aimed to elucidate whether and how virtual reality–based learning influences skill development and attitudes in patient-facing communication.

### Participants

In the pilot test of the course (March – April 2024), enrollment was capped at 12 students, with 11 Master of Science (MSc) or Doctor of Philosophy (PhD) students from the Institute of Medical Science (IMS) participating. For the second offering (September – October 2024), the capacity increased to 30 students, with 29 enrolled. All students were in their first or second year and completed a course approval form due to limited enrollment capacity. For the pilot cohort, students were selected based on their interest in the course and its relevance to their research projects, assessed by the course directors and an IMS staff member. In the second iteration, priority was given to first- and second-year students. Participant consent was implied through enrollment in the course and completion of course activities, as the data collection activities (e.g., completion of VR modules, participation in class debrief, etc.) were embedded within the regular course curriculum and followed the course schedule. Therefore, by participating in the course, students provided implied consent for their data to be used, in accordance with institutional ethical guidelines. Prior to data collection, students were informed about the purpose of data collection (i.e., for course improvement, evaluation, and research) and were given the option to opt out of having their data included in the research analysis. All data were anonymized, and participation had no impact on student grades or standing. A detailed syllabus and course description are available in Online Resource 1.

### VR Modules

This course included two modules on obtaining informed consent using trained actors and investigator-written scripts. Students interacted with the VR system by wearing a headset and holding a controller in each hand to navigate through the scenarios and make selections. Each case presented multiple-choice dialogue options representing possible responses to patient or caregiver statements. Students selected the response they felt was most appropriate, and the case progressed dynamically based on those selections. All responses were pre-determined; free-text or spoken responses were not possible. There were no changes made to the course or VR modules between the first and second offerings.

**Fig. 1:**
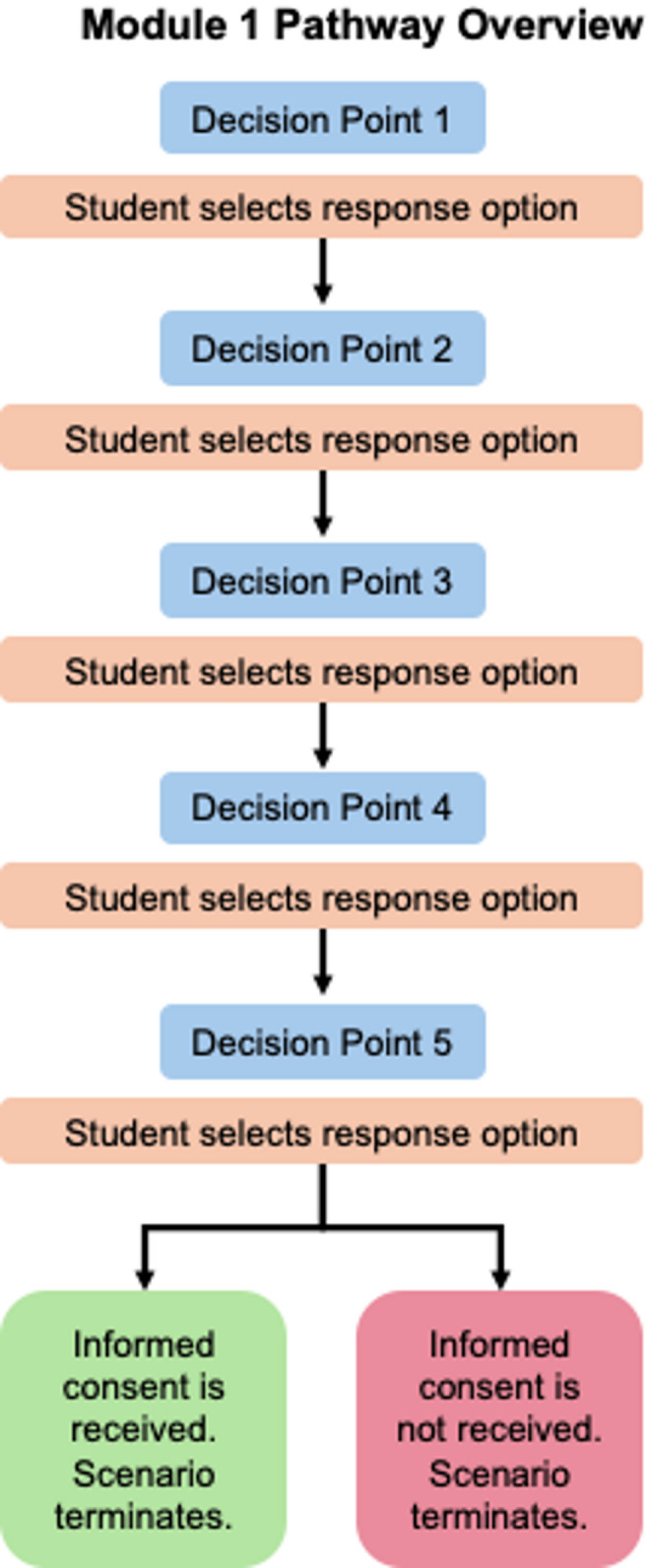
Overview of Module 1 pathway progression

Module 1 focused on fundamental consent practices and followed a linear progression (Fig. 1). Students were tasked with obtaining informed consent from a simulated patient with advanced breast cancer undergoing radiation treatment for brain metastases. The simulated patient was accompanied by a skeptical caregiver who could prevent consent. In the simulation, the student’s research aims to assess the effects of radiation treatment by comparing patient outcomes to those of other treatment methods. As part of the study, participants would undergo tests evaluating cognition, fine motor skills, and memory. Students’ objective for this module was to communicate research aims, objectives, and methods in clear, lay language to the potential participant (simulated patient) while ensuring the patient felt comfortable and fully informed. This VR scenario was developed to complement a lecture series within the course on lay language communication with patients, families, and the public. We chose oncology as the research setting due to its heavy use of medical jargon and complex information. As the first VR module completed by students, it served as an introductory exercise focused on a foundational principle for patient-facing communication, without incorporating additional ethical considerations.

**Fig. 2:**
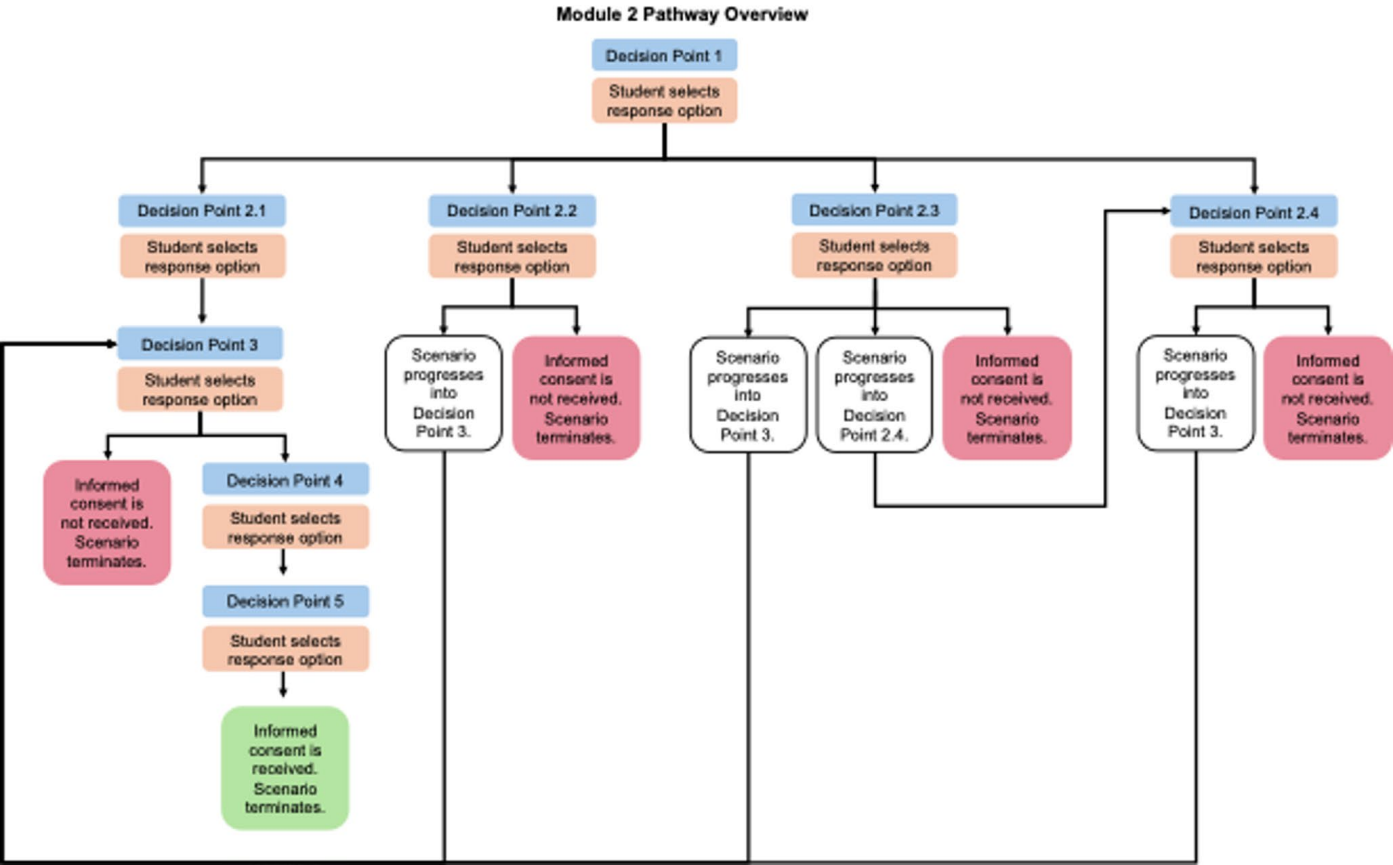
Overview of Module 2 pathway progression

Module 2 introduced a more complex ethical scenario and followed a branching progression (Fig. 2). In this module, students were tasked with obtaining informed consent from an elderly cancer patient with brain metastases and limited English proficiency, accompanied by his granddaughter, who acted as both a cultural broker and translator. This dynamic reflected the expeirences of many immigrant patients who access healthcare services with limited English proficiency [6]. The student’s research aim in Module 2 was the same as that of Module 1, which was to assess the effects of radiation treatment for brain metastases by comparing patient outcomes to those of other treatment methods. As in the previous module, participants would undergo tests evaluating cognition, fine motor skills, and memory. During the simulated interaction, the granddaughter indicated that both she and the potential participant were open to hearing more about the research study and the recruitment process. However, she requested that the diagnosis be withheld - specifically, that terms such as ‘cancer’, ‘tumor’, or any diagnosis-related terminology not be used - citing cultural concerns regarding direct disclosure of the cancer diagnosis. The inclusion of a family caregiver who simultaneously served as a cultural broker and translator was intentional, designed to mirror the lived experiences of immigrant patients and families. Moreover, the decision to incorporate the request for non-disclosure was deliberate, aiming to prompt students to reflect on the balance between ethical disclosure, cultural sensitivity, and patient rights in the context of informed consent. Non-disclosure of diagnoses, particularly in oncology, has been widely used as a case example underscoring the need for cultural nuance and sensitivity in navigating ethical dilemmas in healthcare provision for immigrant populations [7]. In this scenario, we were particularly interested in examining whether students (1) understood the fundamental principles of informed consent; and (2) were able to navigate the situation with nuance, cultural sensitivity, and self-reflection. Therefore, the objective of this module was to serve as an exploratory learning experience, encouraging students to engage critically with the principles of informed consent and to develop the capacity to navigate research interactions with patient participants with nuance, cultural safety, and self-reflection.

While Module 1 reinforced lecture content and served as an acclimatization exercise, Module 2 introduced new ethical dilemmas. Therefore, although students completed both modules, class debriefs primarily focused on Module 2. Although this emphasis was not pre-planned, the debrief sessions appeared to center more heavily on Module 2 due to students’ reactions and engagement with its greater ethical and cultural complexity.

### Data Collection: Quantitative Measures and Data Analysis

All students completed a pre-Knowledge test and ‘Readiness for Clinical Integration’ pre-course survey during the first class and repeated both at the last class (post-course). The Knowledge test included 15 multiple-choice questions (Online Resource 2). Students were not able to access any resources while completing the pre/post Knowledge test. The ‘Readiness for Clinical Integration Survey’, adapted from the Temerty Faculty of Medicine’s MD program, consisted of 6 four-point Likert scale questions and one open-ended question (Online Resource 3). Students also had the option to complete a voluntary course evaluation with 21 five-point Likert scale questions and two open-ended questions (Online Resource 4). Completed tests and questionnaires were not assessed or made available to the course directors and therefore did not affect grades.

The pre- and post-course assessments were designed to evaluate the cumulative impact of the course, with the VR modules positioned as the final experiential component. Students had completed all lectures and group discussions before engaging in the VR activities which were intended to consolidate theoretical learning into applied practice. The pre-course assessments therefore represented students’ baseline knowledge and readiness, whereas the post-course measures reflected growth following both didactic and immersive experiences. Although this design did not isolate the specific contribution of the VR modules, their placement as the course’s primary experiential feature allows the observed gains to be interpreted as evidence of how VR-supported learning can deepen and integrate theoretical instruction.

Data analysis included only completed pre- and post-test sets. Means and standard deviations were calculated and paired sample t-tests were used to compare pre- and post-course scores for the Knowledge test and the Readiness for Clinical Integration Survey using SPSS v26.0, with significance set at *p* <.05. Internal consistency of the Readiness for Clinical Integration Survey was examined using Cronbach’s alpha.

### Data Collection: Qualitative Measures and Data Analysis

In addition to the knowledge and readiness tests and course evaluation, students participated in an in-person debrief and feedback session upon completing the two modules. This session, held during class time, was informal in structure and facilitated by the course director (ET). During the session, students engaged in a class-wide dialogue, providing feedback on the VR modules. They discussed their perceptions of the benefits of the VR experience, the challenges encountered, and any additional thoughts on the content or technical aspects of the VR modules.

Detailed field notes on student responses were taken by NK and used for subsequent analysis. Thematic analysis was conducted by KL and NK [8]. Data from the field notes were coded inductively, line by line. Initial codes were then combined and refined into separate themes on student perspectives and experiences on the technical aspects and student perspectives on the knowledge and content of the modules.

## Results

### Quantitative Findings

#### Knowledge Test Scores

**Fig. 3:**
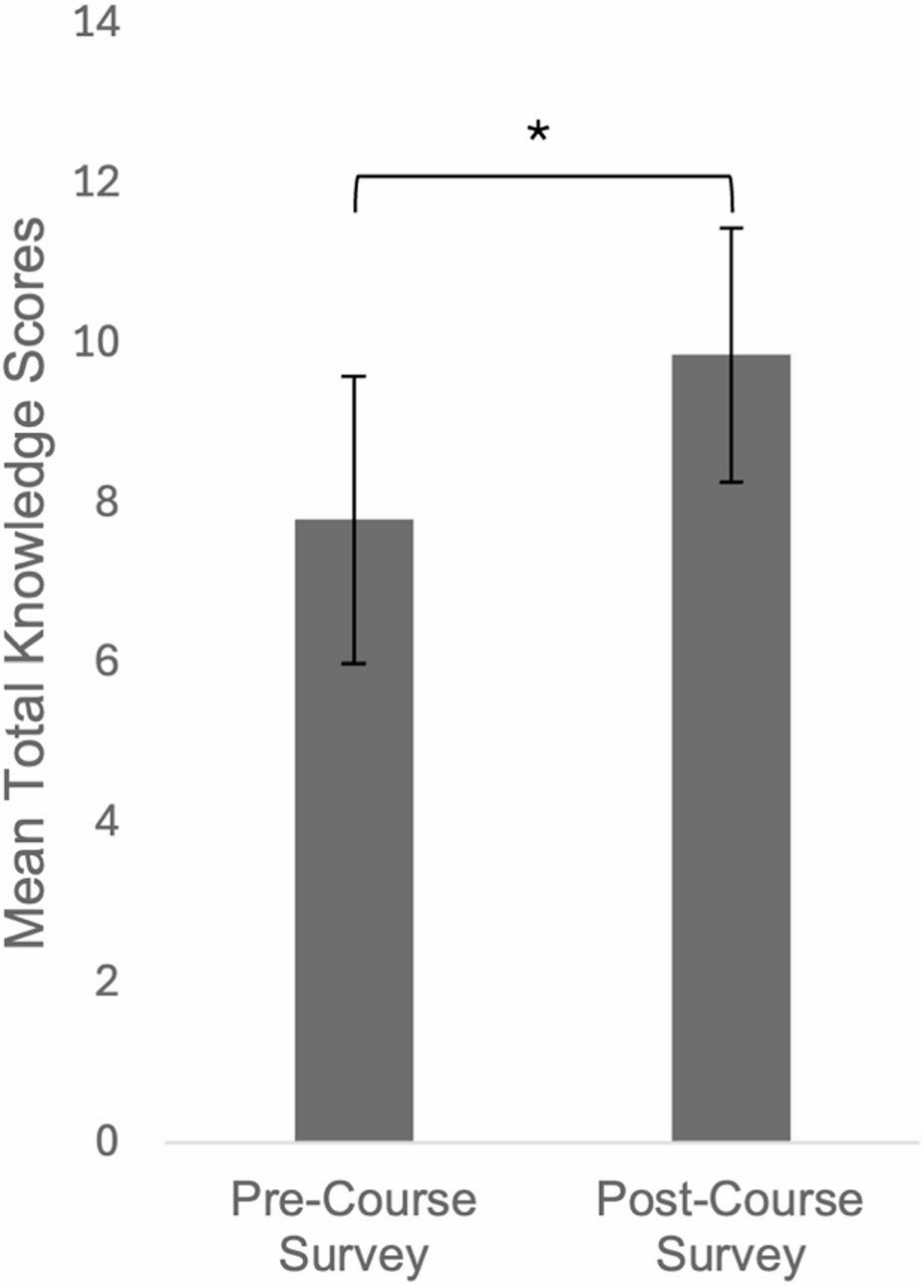
Average pre- and post-course knowledge scores (*N* = 35). Error bars = SD. * *p* <.001

Thirty-five students across both cohorts completed both the pre- and post-Knowledge test. Results indicated a significant increase in post-course scores (M = 9.86, SD = 1.59; Fig. 3) compared to pre-course scores (M = 7.80, SD = 1.80), t(34) = 36.52, *p* <.001, with a large effect size (Cohen’s d = 1.21).

#### Readiness for Clinical Integration Scores

**Fig. 4:**
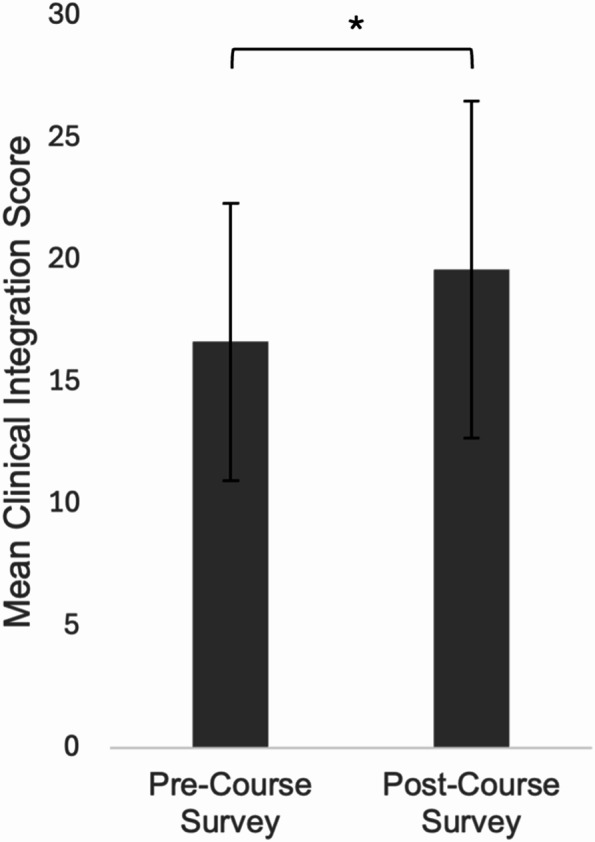
Average pre- and post-course readiness for clinical integration scores (*N* = 40). Error bars = SD. * *p* <.05

Forty students completed both the pre- and post-survey. The Readiness for Clinical Integration Survey demonstrated acceptable internal consistency, with Cronbach’s α = 0.70 pre-course and α = 0.75 post-course. Results demonstrated a significant increase in Readiness for Clinical Integration scores post-course (M = 19.60, SD = 6.91; Fig. 4) compared to pre-course survey responses (M = 16.63, SD = 5.69), t(39) = 5.26, *p* <.05, with a medium effect size (Cohen’s d = 0.47).

#### Module Success Rates

A module was considered successful if the student obtained informed consent from the potential participant; otherwise, it was deemed unsuccessful. For Module 1, only two of eleven students in the pilot course were able to obtain consent from the patient by choosing the ‘best’ response at a critical moment in the scenario. The remaining students (9/11, 82%) chose the ‘second best’ response at that point and were therefore unable to obtain consent. In the second cohort, only four (4/29) students obtained consent from the patient.

In Module 2, four of the eleven students successfully obtained consent from the participant during the pilot course. These students chose to agree to avoid using the term “cancer” at the request of the caregiver, proceeded with the consent process, and ultimately secured consent. In the second cohort of students, nine (9/29) obtained consent from the patient. Of these nine students, five of them inquired whether the caregiver was the legal decision maker and if they could provide legal documentation before obtaining consent. The remaining four students in this group suggested that the family inform the patient about their diagnosis before obtaining consent, citing research indicating better clinical outcomes for informed patients. Of the 20 students who did not obtain consent, three (3/29) students immediately terminated the interaction after concluding that the patient could not participate in the study due to concerns about informed consent.

### Qualitative Findings

Since Module 1 served as an acclimatization module, the qualitative findings from the class-wide debrief session primarily focus on Module 2. A summary of knowledge- and content-related qualitative themes with example quotes is provided in Table 1.

**Table 1:** Summary of knowledge- and content- related qualitative themes with example quotes

Theme	Example Quote
Hesitancy in making decisions during Module 2	*“For the second module*,* it was a difficult choice to make …”*
Difficulty distinguishing researcher vs. clinician roles	*“I would want to consult their chart to see if they are truly unaware [of their cancer diagnosis].”*
Lack of clarity on the principles and purposes of informed consent in research	*“From a consent standpoint*,* there is an issue because you are not fully disclosing the disease.”* *“The whole idea of ethical consent is that they need to understand what the study is*,* what the inclusion criteria is- you can’t just hide parts of the consent process.”*
Beginning to reflect on positionality and worldviews	*“[It’s] too much of a burden on me to remember not to say the word tumor*,* especially if they consent and now [I] have to continuously interact with the patient and always avoid the word”*
Reflecting on the ethical and practical consequences of researcher decisions	*“If I was consenting to a treatment [as a researcher]*,* I would [choose to gain their] consent due to EDI (equity*,* diversity*,* inclusivity) so they don’t miss out on an opportunity… If you don’t consent this patient*,* you’re excluding a population that chooses not to disclose to their family their diagnosis”* *“At first*,* right after the module [2]*,* I was really happy that I didn’t get consent. But then after this conversation [debrief] and [conversation about] EDI*,* I feel like maybe I should have done more work to include them*,* or at least try to.”*

#### Knowledge and Content

Students’ feedback revealed gaps in their understanding of cultural safety and competence. This lack of knowledge led to a hesitation in making decisions within the VR module, as students felt inadequately equipped to navigate culturally safe patient recruitment and informed consent processes.

“For the second module, it was a difficult choice to make …” - Student, paraphrased by NK.

Although students understood the principle of respecting other cultures, they struggled to apply this understanding effectively when making decisions in the module.

Students also struggled to distinguish their roles as researchers versus that of clinicians. The module intentionally created tension by prompting students to reflect on their responsibilities as researchers rather than healthcare providers. While they understood this distinction in theory, they were often uncertain about when and under what conditions they could access a patient’s personal health information for research purposes or disclose medical conditions and diagnoses.

“There was a question…where the granddaughter [caregiver] asked me if a symptom [that the patient had] was normal. I recognized that and had to take myself “out” of that [topic of conversation]” – Student, paraphrased by NK.

Additionally, students expressed concerns about information shared by a patient’s family members or colleagues during the informed consent process. For instance, in Module 2, a caregiver (patient’s granddaughter) requested that the term ‘cancer’ not be used during the recruitment and informed consent process due to cultural reasons. This request created uncertainty among students about their role as researchers, with some considering whether they should consult the patient’s medical chart to verify the caregiver’s statement. Their hesitation stemmed largely from concerns about potential deception in the informed consent process.

“I would want to consult their chart to see if they are truly unaware.” - Student, paraphrased by NK.

“From a consent standpoint, there is an issue because you are not fully disclosing the disease.” - Student, paraphrased by NK.

“The whole idea of ethical consent is that they need to understand what the study is, what the inclusion criteria is- you can’t just hide parts of the consent process.” - Student, paraphrased by NK.

Overall, students found it challenging to set aside their own worldviews to recruit participants and address cultural nuances during informed consent. However, they began to reflect on their own positionality and the implications of paraphrasing eligibility criteria, such as avoiding the use of the term ‘cancer’, during participant recruitment. For example, students conveyed uncertainty about their ability to consistently avoid using the term ‘cancer’, even if they initially succeeded in doing so. This suggested that students began to engage in reflexivity as their own worldviews might make it difficult to maintain this approach over time, leading to apprehension about their accountability in handling cultural nuances for which they felt unprepared.

“How does the patient not know they have cancer?” - Student, paraphrased by NK.

“[It’s] too much of a burden on me to remember not to say the word tumor, especially if they consent and now [I] have to continuously interact with the patient and always avoid the word” - Student, paraphrased by NK.

Furthermore, students began to reflect on the broader implications of choosing to exclude potential participants or populations during the recruitment process. Many expressed concerns about inadvertently disclosing a patient’s diagnosis, either themselves or through other members of the research team, which led to uncertainty and discomfort. Faced with the complexities of recruiting participants with differing worldviews and specific recruitment considerations, some students opted to disengage early, deciding that pursuing informed consent in these cases was “just not worth it”.

However, a class-wide discussion prompted deeper reflection on the ethical and practical consequences of such decisions. Students recognized that avoiding engagement with certain participants due to perceived challenges in recruitment could result in the exclusion of specific populations in an inequitable manner.

“If I was consenting to a treatment [as a researcher], I would [choose to gain their] consent due to EDI (equity, diversity, inclusivity) so they don’t miss out on an opportunity… If you don’t consent this patient, you’re excluding a population that chooses not to disclose to their family their diagnosis” - Student, paraphrased by NK.

Through these discussions, students demonstrated a shift in perspective. They began critically reflecting on their roles and responsibilities as researchers, particularly in distinguishing their duties from those of clinicians. They also emphasized the importance of communicating in plain language with potential participants and reflected on their decisions to exclude certain population groups.

“At first, right after the module [2], I was really happy that I didn’t get consent. But then after this conversation [debrief] and [conversation about] EDI, I feel like maybe I should have done more work to include them, or at least try to.” - Student, paraphrased by NK.

Despite lingering discomfort regarding obtaining informed consent in Module 2, students showed progress in considering alternative worldviews and perspectives. This reflective process encouraged them to practice more culturally safe and equitable approaches to participant recruitment.

#### General Technical Considerations

Students generally agreed that the VR modules were both realistic and immersive. They reported experiencing a heightened sense of urgency due to the presence of virtual patients, who awaited their response selections. This interactive dynamic prompted students to dedicate more time and thought to their decisions compared to more traditional formats, such as pen-and-paper exercises or computer modules, which they admitted might otherwise encourage rapid, superficial engagement solely to complete the activity.

The VR technology was noted to enhance knowledge acquisition by offering immediate feedback and opportunities to retry the modules. However, students highlighted an initial adjustment period as they acclimated to the VR environment. Early sessions were marked by disorientation, which they attributed to issues such as height calibration within the system. This disorientation momentarily distracted them from focusing fully on the module content. Despite these initial challenges, students reported that, once they adapted, the VR system was relatively intuitive and user-friendly.

## Discussion

Virtual reality (VR) has emerged as a promising educational tool in health education, gaining significant attention since the COVID-19 pandemic for its safety, cost-effectiveness, and efficiency [9]. In this study, we evaluated the impact of VR-based modules on graduate students’ ability to navigate communication challenges, ethical patient-facing dilemmas, and culturally safe scenarios within a clinical research context. Our results emphasize VR’s potential as a comprehensive and accessible educational tool to foster non-technical skills that are critical for effective clinical research.

### VR’s Role in Teaching Non-Technical Skills

In our study, VR modules provided graduate students with a safe and controlled environment to practice complex research communication, ethical decision-making, and culturally safe interactions. Module 1 emphasized the delivery of complex medical information using professional and clear language, such as explaining a neurocognitive test. Module 2, on the other hand, focused on ethical dilemmas and cultural competency, enabling students to reflect upon inclusivity and equity in research practices. These modules immersed students in realistic scenarios to allow them to explore nuanced challenges and refine their communication strategies without the fear of real-world repercussions.

Our findings add to the growing body of evidence suggesting that VR can be effective in teaching non-technical skills such as empathy, situational awareness, and communication [10]. For example, one study found that undergraduate students trained with VR were better prepared for real-world patient interactions, including obtaining consent and responding to patient questions in a more accurate and timely manner [11]. Additionally, VR has been shown to help students reflect on their emotions and behaviors, enhancing their ability to navigate future patient interactions [12]. The emotional impact of health education training using VR on self-efficacy has been noted in other studies [13], and trainees often appreciate the opportunity to experience interactions with others in problematic or stressful contexts [14]. While our surveys spanned the semester as opposed to pre- and post-VR modules, our results suggest that VR-based learning enhances students’ confidence and preparedness for real-world clinical scenarios. These findings align with prior research indicating that immersive learning environments can enhance both cognitive and practical skills [10, 11].

VR further demonstrates potential as a sustainable method for teaching non-technical clinical skills, such as communication. Traditionally, these skills are taught using standardized patients (SPs) - trained individuals who portray patients or family members in scripted scenarios with specific historical, emotional, or physical characteristics [15, 16]. Unlike peer role-play, where learners act out roles themselves, SPs are professionally trained to respond, engage, and provide feedback in a standardized manner, minimizing variability between student encounters [15–17]. The cost of using SPs in medical education varies by region and institutional policies. In Ontario universities, SPs typically earn $21–27 an hour, depending on the institution and project [18–21]. Despite these costs, the use of SPs has been shown to be more cost-effective compared to traditional role-playing, case exercises, or traditional didactic lecture formats in teaching communication skills across health disciplines [22–25]. However, even with standardized training, variability among SPs and learners remains inevitable, prompting the development of scales to assess SP performance to maintain consistency [26]. VR offers comparable training quality to SP-based instruction while addressing key challenges such as variability and scalability by providing highly consistent and repeatable learning experiences. For instance, undergraduate nursing students reported that VR simulations fostered a similar level of emotional engagement as SP encounters, supporting the development of effective communication skills [27]. VR also enables educators to “spread” (train more students) and “scale” (develop new scenarios for different learning objectives) their teaching efforts [28]. Although VR implementation may require substantial upfront investment in technological hardware and content development, it can ultimately serve as a more sustainable and cost-effective approach in the long term. Evidence from other areas of medical education, such as evacuation training, supports this long-term cost efficiency [29]. Thus, while VR may not yield immediate financial benefits, sustained use can generate a higher return on investment for teaching non-technical clinical skills in medical education.

### Students’ Performance in VR and Identified Areas for Improvement

Overall, students reported satisfaction with the VR module and valued the opportunity to practice interacting and communicating with virtual patients in an immersive, low risk setting. Although students demonstrated a commendable ability to select the most appropriate responses in various scenarios during the VR modules, there were several moments during these modules that signaled a need for enhanced training in patient communication.

In Module 1, many students faced challenges in simplifying the neurocognitive test into lay language that would be easily understood by potential study participants (Data not shown), further evidenced by the low success rates for obtaining informed consent. To obtain informed consent, students were required to select the best response for at least four of five decision points. Across both cohorts, most students demonstrated proficiency in explaining medical jargon and addressing privacy and confidentiality (Cohort 1: 25/29; Cohort 2: 12/12). However, many struggled to describe the research test, specifically, in describing its purpose and procedures in accessible, lay language, with most selecting either the second-best or least appropriate response (Cohort 1: 19/29, Cohort 2: 8/12). These findings suggest that students are more comfortable translating technical or content-based information into lay language but have greater difficulty in communicating procedural aspects of research participation. Some students, particularly in the second cohort, also struggled to describe participation risks. In several cases, they selected responses that combined potential risks with possible benefits, which may reflect an attempt to reassure participants but risks conflating risk disclosure with persuasion. Overall, these results highlight specific areas, particularly communication about research procedures and risks, where additional instructional emphasis in the course may be needed to enhance students’ ability to obtain informed consent. The low success rates also emphasize the need for targeted training in patient-centered science communication that is understandable and accessible to the general public. Calls for such training are not new; previous literature has underscored the importance of making scientific concepts accessible to the general population [30–32]. For example, the Canadian Institutes of Health Research, the federal agency responsible for health and medical research in Canada, states that a central premise of knowledge translation and research dissemination, such as science communication, should be conveyed in a way that is relevant and useful to knowledge users [33]. Addressing this gap is crucial in improving patients’ and potential research participants’ comprehension of study procedures and facilitating more informed decision-making in research contexts.

In Module 2, students encountered an ethical dilemma during the informed consent process when asked to avoid the term ‘cancer’. To obtain informed consent, students needed to select the best or second-best response at the first decision point, in which a caregiver requested that the diagnosis not be disclosed. Alternate outcome pathways were designed to prompt reflection on related ethical issues, including the legal implications of non-disclosure, whether a participant can meaningfully consent without full knowledge of their diagnosis, and the distinction between the roles of researcher and clinician. Most students were unable to obtain informed consent in Module 2 (Cohort 1: 7/11; Cohort 2: 20/29), largely due to their initial response. Qualitative findings suggest that students’ struggle to balance cultural safety with full disclosure contributed to low Module 2 success rates, as many feared that omitting diagnostic terms would compromise the validity of informed consent and constitute deception. However, deception in research typically involves deliberately misleading participants, often to reduce bias [34]. The purpose of informed consent in research is to ensure that participants fully understand the study’s purpose, risks, benefits, procedures, and how their data will be used before agreeing to participate [35]. In Module 2, acknowledging the cancer diagnosis was not necessary for participation, as the simulated study focused on cognitive and motor outcomes of radiation therapy. This made disclosure of the diagnosis irrelevant to both the study’s validity and the ethical standard of informed consent. Students’ discomfort with recruiting the simulated patient without using diagnosis-related terms highlight the need for more comprehensive education on research ethics and informed consent, particularly regarding the principles and purposes of informed consent.

Students also reflected on the implications of their personal worldviews, recognizing how their biases may influence participant inclusion. This finding is notable, as researchers who fail to identify or suspend their personal biases may exclude participants from certain demographics, undermining efforts to achieve equity, diversity, and inclusion within a research setting. Encouraging such reflections is essential for fostering inclusivity and equity in participant recruitment. To further develop students’ cultural safety skills, a transformative educational approach may be beneficial. Cultural safety fundamentally requires self-reflection; in research contexts, it involves engaging with participants in ways that respect their social, political, linguistic, economic, and spiritual realities [36]. Cultivating these skills often entails rethinking how knowledge is understood, valued, and produced, which are often facilitated through transformative learning. Transformative learning is characterized by the revision of assumptions and expectations to become more inclusive, open, reflective, and adaptable [37]. When learners encounter perspectives that challenge their existing beliefs, they are prompted to critically examine their underlying assumptions [37]. This process often begins with a “disorienting dilemma” (an uncomfortable situation) that disrupts established ways of thinking [38], much like the ethical challenge presented in Module 2. Although we did not assess students’ cultural safety skills before and after Module 2, our qualitative findings suggest that the module served as a transformative experience, prompting critical reflection and potentially enhancing cultural safety competencies. Continued engagement in transformative learning experiences is likely necessary for students to develop greater confidence and competence in culturally safe research practices.

We believe that the depth and emotional resonance of students’ responses in Module 2, which resulted in these findings, were in large part facilitated by the use of virtual reality. The immersive nature of the VR experience may have contributed to more dynamic engagement and provoked deeper reflection during the debrief session. We are uncertain whether a comparable level of emotional and ethical complexity would have emerged had the module been delivered through a more traditional format, such as a text-based case studies or standard computer-based simulations.

While our VR module served as an effective starting point for these reflections, transformative learning requires repeated exposure and deeper engagement with such frameworks. These findings underscore the need for stronger emphasis on ethical communication and culturally safe research practices in research training programs. Equipping research trainees with the skills to navigate diverse cultural perspectives will help them uphold ethical standards while fostering respectful and inclusive research environments.

### Study Limitations

This study has several limitations. First, the lack of a control group limits the ability to attribute improvements in students’ confidence, preparedness, and knowledge solely to the VR intervention. Future research with randomized controlled designs comparing traditional training or no intervention is recommended. Second, a small sample size reduces generalizability, and reliance on self-reported data may introduce response bias, especially as questionnaires included student names. Further, self-perceived gains may not reflect actual skill or knowledge improvements, highlighting the need for objective assessments like standardized tests, peer evaluations, or faculty observations. Third, because the pre- and post-course assessments captured cumulative learning from the entire course rather than changes immediately before and after the VR modules, it was not possible to disentangle the unique impact of the VR modules to observed improvements. The pre-course surveys were administered at the start of the semester prior to any lectures or discussions, while the VR activities occurred near the end of the course. As a result, the gains that occurred post-course likely reflect the combined influence of both didactic instruction and experiential learning from completion of the VR modules. Fourth, since data were collected across two academic terms, cohort differences and timing effects may have influenced outcomes. Fifth, our findings may have been confounded by variation in students’ prior research experience. Although the course primarily enrolled first- and second-year graduate students, it is possible that some students had previous professional or academic experience, which may have influenced their baseline readiness and learning gains. Sixth, no qualitative data was gathered for Module 1, as it served as an acclimatization module. Consequently, findings primarily reflect experiences from Module 2, which emphasized equity, diversity, and inclusivity. Finally, this study assessed short-term outcomes without exploring long-term skill and knowledge retention. Future research should investigate whether VR learning translates to lasting improvements in clinical or research performance.

### Future Directions

VR offers a unique opportunity for students to practice non-technical skills clinical research skills like patient-centered communication, ethical decision-making, and cultural safety in realistic, low-risk scenarios. While preliminary, these findings suggest opportunities to refine and expand VR’s educational potential.

VR’s immersiveness and adaptability make it ideal for diverse settings – students may feel more immersed particularly in settings that are emotionally charged, such as in Module 2. Future studies may explore the conditions under which virtual reality offers advantages over traditional or computer-based teaching methods. Future modules could simulate interdisciplinary team environments, fostering collaboration among researchers, clinicians, allied health professionals, and knowledge translation specialists. These experiences could prepare trainees for real-world decision-making in modern healthcare. VR could also introduce undergraduate medical science students to ethical dilemmas or support continuing education for practitioners or emerging practices.

Though our study did not evaluate the cost-savings of using VR, it did demonstrate that VR can be effective in teaching non-technical clinical skills in medical science education. Future work may further explore the comparative cost-effectiveness and long-term sustainability of VR- versus alternate, more traditional approaches (such as SP-based approaches, role-playing, case studies, or didactic formats) for teaching non-technical clinical skills to graduate medical science students.

In conclusion, VR’s flexibility and engaging nature position it as a transformative tool in healthcare education, capable of enhancing collaborative learning, tackling systemic challenges, and supporting ongoing education for both emerging and experienced professionals.

## Supplementary Information

Below is the link to the electronic supplementary material.

Supplementary Material 1.

Supplementary Material 2.

Supplementary Material 3.

Supplementary Material 4.

Supplementary Material 5.

## Data Availability

The datasets generated during and/or analyzed during the current study are available from the corresponding author on reasonable request.
