# Supplementary material for: Virtual Reality for Developing Patient-facing Communication Skills in a Medical Science Graduate Education Course: A Mixed-Methods Pre-Post Study
Source: Med Sci Educ. 2025 Dec 29;36(1):201–12. doi: 10.1007/s40670-025-02604-4 (PMC13043831; doi:10.1007/s40670-025-02604-4)
Supplement: Supplementary file 1 — Supplementary Material 1. [file 40670_2025_2604_MOESM1_ESM.docx]

Virtual Reality for Developing Patient-Facing Communication Skills in a Medical Science Graduate Education Course: A Mixed-Methods Pre-Post Study

Authors: Kyla Gaeul Lee, Maryam Sorkhou, Nicole Harnett, Sobiga Vyravanathan, Theodore J. Brown, Evan Tannenbaum, Nairy Khodabakhshian*

*Corresponding Author: Nairy Khodabakhshian

Institute of Medical Science, C. David Naylor Building, University of Toronto

6 Queen’s Park Crescent, Suite 119, M5S 3H2, Toronto, Canada

nairy.khodabakhshian@mail.utoronto.ca

**MSC1121H: Clinical Research Skills – Course Syllabus and Outline**

**Course Director: TBA**

**Sessional Lecturer: TBA**

**Drop Date: TBD**

**GENERAL**

**Target Audience:** Graduate students at the Institute of Medical Science (IMS) conducting graduate research with a clinical focus. Priority will be given to graduate students who will be or are conducting clinical research who are patient-facing. However, all students who are interested in gaining clinical skills relevant to research are welcome to apply.

**Course Description:**

This is a mixed-media course designed to educate and familiarize graduate students to the uniqueness of the clinical environment to support their clinically-focused graduate research. Completing this course before physically integrating into the live environment will help facilitate a smoother transition into the clinical space and team for students. Currently at IMS, graduate students are expected to self-learn the multifaceted skills needed to succeed in the clinical environment. This course aims to fill the gap by providing graduate students with a formal learning environment using immersive virtual reality and traditional learning methods (ie. lectures, readings, discussion) to gain skills that will support clinical and health services research.

Graduate students who are conducting clinical research, health services research, or are patient-facing will benefit from this course. In this course, students will learn how best to navigate inter- and multi-professional teams within the clinical setting. Students will also learn how to communicate with patients and their caregivers to recruit, educate, support, and maintain relationships with patients participating in their studies. Specific activities include, but are not limited to, obtaining informed consent, communicating study objectives in lay language, ethical practices in research with patient participants, confidentiality, and EDI in research with patient participants. This will be a particularly useful course for graduate students who are new to conducting patient-facing and/or clinically-focused graduate research.

**Course Objectives:**

The primary goals of this course include:

1. Use innovative technology (e.g. immersive simulated experiences/virtual reality) that accurately depict the clinical environment to build initial skills for interacting with patients and their caregivers/decision makers including, but not limited to:
   1. Communicating using plain language to ensure patient/caregiver understanding,
   2. The ethics related to and steps for recruiting patients to clinical research,
   3. Having challenging conversations regarding clinical research,
   4. Maintaining professionalism in the clinical setting.
2. Orienting the student to the clinical setting (vs. academic setting) to prepare them for the
   1. culture of the clinical environment including, but not limited to:
   2. Working as part of a multidisciplinary health care team,
   3. Navigating a patient-focused clinical environment,
   4. Managing professional relationships in the clinical setting.

**Learning Objectives:**

By the end of this course, students will be able to:

1. Effectively communicate with patients, caregivers, and decision-makers in simple, clear, and plain language.
2. Identify techniques and elements that support patient engagement throughout the research process.
3. Work as part of a multidisciplinary health care team including but not limited to working with physicians, nurses, researchers, social workers, physiotherapists, technicians, etc.
4. Navigate the clinical environment ethically and professionally.
5. Ensure adherence to ethical clinical research practices including patient consent, privacy, and data integrity protocols.
6. Describe the ways in which equity, diversity, and inclusion can be reflected in the research process, including but not limited to during the process of patient recruitment, informed consent, collection of data, patient engagement, debriefing, and dissemination of results.

**Requirements:**

- Students who are planning to, interested in, or currently conducting clinical research for a
- graduate degree.
- There are no prerequisites and exclusions for this course.
- Pre-approval for this course is required. Students will have to contact the course instructor and provide a brief statement of interest.
  - “Please send a brief statement of interest/background (no longer than 300 words) via email to the course instructor for enrollment approval."
- Attendance is mandatory unless absence is arranged/approved in advance by course faculty.
- Make-up sessions may need to be scheduled.

**Course Materials**

Students will require access to University of Toronto’s Quercus portal to access readings and

Instructions.

**SCHEDULE**

**Format:**

This course is a 6-week, 0.25 FCE course running from x to y. Weekly sessions take place every

[weekday], from [time-time] and consist of:

- Didactic Lectures covering majority of course content
- Active learning for remaining course content and via case-study presentation evaluation
- Virtual reality for practical application of course content

**Weeks 1 & 2: Plain Language Communication**

Topics will include: Theory and significance, equity and principles, techniques, informed consent and dissemination, patient-centered plain language communication

Hands on: Assignment

**Weeks 3 & 4: Research Ethics**

Topics will include: Confidentiality, informed consent, anonymization, collecting sensitive data. Week 3 will cover the Research Ethics application process and elements to consider, while week 4 will incorporate active learning through case study examples.

Hands on: Case Studies

**Weeks 5 & 6: Professionalism**

Topics will include: Researcher’s roles and expectations, working in interprofessional clinical settings and teams, having difficult conversations, professional communications, plain language communication

Hands on: Role Playing, Virtual Reality

**Evaluation: CR/NCR. A 70% will be required to obtain a credit for the course.**

1. Class Participation & Student Engagement (Pass/Fail)
2. Virtual Reality
   1. Part A: VR Completion (Pass/Fail)
   2. Part B: Pre- and Post- Knowledge Questionnaire (Pass/Fail)
   3. Part C: VR Content Feedback Questionnaire (Pass/Fail)
3. Case Study Presentation (Pass/Fail)

**Evaluation Item #1: Class Participation & Student Engagement:**

- This evaluation component will address Course Objectives 1a-d and 2a-c and Learning Objectives 1, 3, and 6.
- Students will receive full marks for attending all 6 classes. Attendance is mandatory unless pre-arranged with course faculty. Make up sessions may be required to ensure skill acquisition.
- Student engagement grades will be assigned based on the level of student participation throughout the entirety of the course. The assigned grade will reflect the degree of reflection conducted by the student on course content, insightful contributions to discussion, and exchange, produce, and critique ideas as active participants of the learning process. Students are encouraged to prepare comments and responses before class time, and consider the level of thoughtfulness and depth in their responses to others during class time, also reflecting professionalism within a multidisciplinary academic setting. For example, students may initiate discussions with questions or comments of their own, practice “thinking out loud”, and connect ideas with earlier readings or readings outside of the scope of the course. Engaged and participating students will practice patient-focused thinking in their questions and discussions, and work together with peers to reflect development of professional relationships.

| **Grade** | **Pass** | **Fail** |
| --- | --- | --- |
| **Description** | 1. Students will attend all 6 classes unless pre-arranged with course faculty. 2. Students will show preparedness by completing all assigned readings and taking notes or being prepared in advance. 3. Students will prepare comments and questions before class time. 4. Students will provide thoughtful and deep responses to others during class time. 5. Students will initiate discussions with questions or comments of their own and practice “thinking out loud.” 6. Students will connect ideas with earlier readings or readings from outside the scope of the course (e.g. other literature, case studies, personal experiences and anecdotes). 7. Students will practice patient-focused thinking in their questions and discussions and collaborate effectively with others in the class. | 1. Student does not attend all 6 classes and/or does not attempt to pre-arrange with course faculty. 2. Student fails to take part in class discussions, does not engage with other students or with the instructor if/when called upon. 3. Student responds with little thoughtfulness, reflection, comprehension of course content, and/or willingness to engage with course content or the ideas of others. 4. Student rarely initiates discussion or actively takes part in class discussion unless they are called upon. 5. Student fails to answer in part or in full, usually because they have not reflected on course content before the class and/or failed to complete reading assignments. 6. The student may be physically in the class, but is distracted with other materials that are not relevant to the course (e.g. doing other work on their laptop). |

**Evaluation Item #2: Successful completion and assessment of VR**

- This evaluation component, consisting of the VR completion, knowledge questionnaire, and VR usability feedback, will address Course Objectives 1a-d and 2a-c and Learning Objectives 1-6.
- Prior to commencing the course, students will be required to complete a questionnaire to determine their baseline clinical research skills knowledge (pre- and post-knowledge questionnaire). This questionnaire will be re-administered at the end of the course to determine whether course objectives were successfully met, whether knowledge gaps were filled, and to provide students an opportunity to present feedback for subsequent iterations of the course. Students will receive a “pass” if both pre- and post-course questionnaires are completed and submitted in full with insightful comments.
- Students will also be required to undergo the pilot virtual reality (VR) experience (physically wear the VR headset, undergo the VR scenario) and provide their feedback on the VR content including, but not limited to, the comfortability and usability of the experience, success in addressing the course and learning objectives, other suggestions for user experience improvement, and modifications required for future semesters. Students will be encouraged to comment on the patient profile, decision-making points, and other relevant aspects of the VR scenario(s) as it relates to the course content. Students will receive a “pass” for completing the VR and submitting insightful written feedback on both the content and the delivery of the VR.
- Assessments will be marked by the TAs. Rubrics will be required.

| **Grade** | **Pass** | **Fail** |
| --- | --- | --- |
| **Description** | 1. Student has attempted and/or completed the VR in its entirety, or up until the point tolerated by the student. 2. Student has completed the pre-post knowledge questionnaire and provided meaningful insight. For example, successful students will reflect on how the level of understanding of the course content has developed and changed at the end of the course compared to the beginning. 3. Student has completed the VR questionnaire in its entirety. Student has provided insightful and detailed feedback such as, but not limited to, the user-friendliness of the VR, comfortability, relevance of the VR content, delivery of the VR content, ability to follow the plot, relevance of decision-points, and overall experience. 4. The exceptional student will provide deep and insightful feedback on the content of the VR scenario by commenting on how the content can be improved. For example, such students may suggest alternative decision-making points, improvements for diversity of the patient profiles, and suggest alternative cases for future incorporation into the VR scenarios. | 1. Student has not attempted to complete the VR. 2. Student has not completed the pre- and post-knowledge questionnaire and/or has provided minimal to no insight/feedback. 3. Student has not completed the VR content questionnaire. Student has provided minimal (i.e. 1-2 brief sentences) or no feedback on the VR experience, relevance, patient profile, decision- making points, and overall usability and comfortability of the experience. 4. The student does not reflect on the content of the VR scenario in the VR questionnaire, but rather solely provides feedback on usability and feasibility of the program. |

**Evaluation Item #3: Case Study Presentation**

- This evaluation component will address Course Objectives 1a-d and 2a-c and Learning Objectives 1-6.
- Students will be provided with a case study problem that incorporates elements of either plain language communication, ethics, or professionalism. Student groups will receive one grade (i.e. partners will receive one grade).
- Students will partner with one or two other students in the course (who have a sufficiently different research background) to create a 10 minute presentation on how they would approach the case study problem. Potential case study problems may include, but are not limited to, obtaining research consent from an individual who does not speak English and maintaining clinical trial participation for individuals with a substitute decision-maker.
- Presentations will be given at the beginning of each class before course content is delivered by the instructor.
- Presenting students will not be graded on the “correctness” of their approach to the case study, but rather, on the degree of insight in their approach. For example, students should explicitly justify their rationale for their approach in the case study.
- Presenting students will incorporate active learning into their presentation to meaningfully engage other students in their presentation. For example, presenting students may initiate discussions with the rest of the class during the presentation, and/or use technology to engage students (e.g. kahoot, word clouds)
- Students will also be graded on their participation with presenting students’ case study presentation by assessing their degree of engagement, answering questions, providing insightful comments, and participation in their presentation

| **Grade** | **Pass** | **Fail** |
| --- | --- | --- |
| **Description** | 1. The presenting student(s) will adhere to the presentation and/or Q&A time limit. 2. The presenting student(s) will effectively collaborate with partners/peers to complete the assigned case study. 3. The presenting student(s) presents their case study analysis with sufficient insight and justification (rationale) in their approach to handling the case study 4. The presenting student(s) uses active learning approaches to meaningfully engage the class and attendees in their presentation. 5. The presenting student(s) incorporate at least 2 class discussion topics to their presentation. 6. Attending students are meaningfully engaged in their peers’ presentations by actively asking questions, answering questions, offering comments, and engaging with the active learning material. 7. Attending students complete peer feedback forms and provide insightful and descriptive feedback to colleagues’ presentations. discussion topics to their presentation. | 1. The presenting student(s) do not adhere to the presentation and/or Q&A time limit. 2. The presenting student(s) do not make sufficient effort to collaborate with partners/peers to complete the assigned case study. 3. The presenting student(s) present their case study analysis without sufficient insight or justification (rationale) in their approach to handling the case study. 4. The presenting student(s) does not use active learning approaches to meaningfully engage the class. For example the presenting student(s) may read off their powerpoint slides in a didactic-lecture style instead of meaningfully engaging the class in discussion. 5. The presenting student(s) incorporate minimal/no class6. Attending students are not meaningfully engaged in their peers’ presentations. Such students may not answer questions, not offer comments, and be unwilling to engage with the active learning material. 6. Attending students do not complete peer feedback forms and/or do not provide insightful and descriptive feedback to colleagues’ presentations. |

**Attendance Policy**

Attendance is mandatory unless pre-arranged with course faculty. Make up sessions may be required

to ensure skill acquisition.

**Instructor Policies**

Communication with the instructor (office hours: office location, dates and times availability, email,

what to do if no response, etc)

Expectations around classroom/online participation and assessments (late/penalty, exceptions, etc)

**Resources**

- Academic Consideration
- Student accessibility services● Writing support
- Mental health resources

**University Policies**

- Academic Offenses
- Use of electronic devices
- Examinations
- Assessment submissions
- Support services
- Student absences
- Registrar
