# Supplementary material for: Virtual Reality for Developing Patient-facing Communication Skills in a Medical Science Graduate Education Course: A Mixed-Methods Pre-Post Study
Source: Med Sci Educ. 2025 Dec 29;36(1):201–12. doi: 10.1007/s40670-025-02604-4 (PMC13043831; doi:10.1007/s40670-025-02604-4)
Supplement: Supplementary file 2 — Supplementary Material 2. [file 40670_2025_2604_MOESM2_ESM.docx]

Virtual Reality for Developing Patient-Facing Communication Skills in a Medical Science Graduate Education Course: A Mixed-Methods Pre-Post Study

Authors: Kyla Gaeul Lee, Maryam Sorkhou, Nicole Harnett, Sobiga Vyravanathan, Theodore J. Brown, Evan Tannenbaum, Nairy Khodabakhshian*

*Corresponding Author: Nairy Khodabakhshian

Institute of Medical Science, C. David Naylor Building, University of Toronto

6 Queen’s Park Crescent, Suite 119, M5S 3H2, Toronto, Canada

nairy.khodabakhshian@mail.utoronto.ca

**Pre/post knowledge**

Name: _____________________________

Please circle the correct answers.

1. What is the prevalence of low health literacy in Canada?

a) 20%

b) 30%

c) 50%

d) 60%

2. The impacts of low health literacy includes all of the following except:

a) Less use of preventative health behaviours

b) Less anxiety and distress

c) Delayed Diagnoses

d) Less adherence to medical instructions

e) Higher costs to the health system

3. Plain language editing techniques include all of the following except:

a) Break complex information into understandable chunks

b) Use simple language or define technical terms

c) Provide ample white space so pages look easy to read

d) Use short sentences

e) Writing less precisely

4. Readability assessments involve all of the following except:

a) The vocabulary used in a text (assessed by syllable counts)

b) The number of words in a sentence

c) The presence of definitions of medical jargon

d) The number of sentences in a paragraph

5. What are some common sources of conflict in the clinical workplace

a) Personal differences

b) Environmental stress

c) Role incompatibility

d) All of the above

6. Conflict navigation with the CRIB method involves all of the following except:

a) Committing to find a new way forward together

b) Reflecting on each other's motivations

c) Independent strategy formation

d) Brainstorming new avenues

7. You experience conflict in the workplace. One of the clinicians raises their voice at you and says: "stop slowing down my clinic!". What is the best next step:

a) Confront the clinician and ask them exactly how you are slowing down the clinic

b) Leave the clinical space immediately, inform your supervisor that you refuse to work with that person in the future, and report the clinician to human resources.

c) Acknowledge the comment and mention that you will speak to your supervisor. While speaking with your supervisor, you mention that you want to work on being more efficient in order to avoid slowing down clinical workflow.

d) Acknowledge the comment and mention that you will speak to your supervisor. While speaking with your supervisor, mention that you felt uncomfortable being spoken to like that and ask what they think you should do.

8. How does the REB define “minimal risk” research?

a) Research in which there are no medical harms to participants.

b) Research in which the likelihood of possible harms related to participation are no greater than those encountered by participants in their everyday lives.

c) Research in which the likelihood of possible harms is less than the likelihood of possible benefits.

d) Research in which there are no medical, psychological or social harms to participants, and where the likelihood of possible benefit to participants is also absent.

e) None of the above.

9. In Canada, which of the following research requires REB review:

a) Research involving living human participants.

b) Research involving human biological materials, as well as human embryos, fetuses, fetal tissue, reproductive materials, and stem cells. This applies to materials derived from living and deceased individuals.

c) Research involving living non-human animals.

d) Both a and b.

e) All of the above.

10. “Risk” is defined as:

a) The absence of benefit.

b) The magnitude or seriousness of the harm.

c) The probability of occurrence of the harm.

d) Both b and c.

e) None of the above.

11. The term “clinical equipoise” refers to:

a) Equal access to participation in clinical research.

b) The use of a placebo in a randomized controlled clinical trial to maintain blinding.

c) Uncertainty about what interventions are most effective for a given condition.

d) Both a and c.

e) None of the above.

12. Which of the following are exempt from REB review?

a) Quality assurance and quality improvement projects which are exclusively for assessment, management, or improvement purposes.

b) Pilot studies to assess the feasibility of a subsequent study, which are not intended to produce definitive results with regard to the research question.

c) Using tissue from deceased individuals for research purposes.

d) Both b and c.

e) None of the above.

13. Which of the following guide Human Research Ethics Boards in Canada?

a) Assisted Human Reproduction Act

b) Tri-council Policy statement

c) Privacy Legislation

d) All of the above

e) Both b and c

1. Which of the following statements is true?

a) Research Ethics Boards consider the scientific aspects of a study

b) Once consented to a study, it is not necessary to inform study participants of new information that might affect their decision to participate.

c) Offering large monetary incentives for participation in research studies is an acceptable way to increase study enrollment

d) Minor revisions to a consent form do not require research ethics board approval

e) Human Research Ethics Boards were established as a result of the Nuremberg Trials at the end of World War 2.

1. Which of the following required the formation of formal Institutional Human Research Ethics Boards in the U.S.?

a) The declaration of Helsinki

b) The Nuremberg Code

c) The Belmont Report

d) The Common Rule

e) All of the above
