# Supplementary material for: Virtual Reality for Developing Patient-facing Communication Skills in a Medical Science Graduate Education Course: A Mixed-Methods Pre-Post Study
Source: Med Sci Educ. 2025 Dec 29;36(1):201–12. doi: 10.1007/s40670-025-02604-4 (PMC13043831; doi:10.1007/s40670-025-02604-4)
Supplement: Supplementary file 3 — Supplementary Material 3. [file 40670_2025_2604_MOESM3_ESM.docx]

Virtual Reality for Developing Patient-Facing Communication Skills in a Medical Science Graduate Education Course: A Mixed-Methods Pre-Post Study

Authors: Kyla Gaeul Lee, Maryam Sorkhou, Nicole Harnett, Sobiga Vyravanathan, Theodore J. Brown, Evan Tannenbaum, Nairy Khodabakhshian*

*Corresponding Author: Nairy Khodabakhshian

Institute of Medical Science, C. David Naylor Building, University of Toronto

6 Queen’s Park Crescent, Suite 119, M5S 3H2, Toronto, Canada

[nairy.khodabakhshian@mail.utoronto.ca](mailto:nairy.khodabakhshian@mail.utoronto.ca)

**CLINICAL RESEARCH SKILLS – MSC1121H**

**READINESS* FOR CLINICAL INTEGRATION PRE-COURSE SURVEY**

Integrating into the clinical environment for the first time can be daunting for a new researcher. In this course, we aim to provide you with the basic knowledge, skills and tools necessary to begin your journey in clinical research where you will engage with real patients and their family/support networks, as well as with health care team that cares for them.

**The term ‘readiness’ infers your level of preparedness and how capable you feel. It is not a measure of your enthusiasm or eagerness.*

The following questions aim to evaluate your readiness to engage in the clinical research setting.

1. Please rate your level of readiness for the following:

|  | 1 – not at all ready | 2 – not very ready | 3 - fairly ready | 4 – totally ready |
| --- | --- | --- | --- | --- |
| Interact respectfully with patients and their family/support network in the conduct of your research. |  |  |  |  |
| Communicate effectively with patients and their family/support network in the conduct of your research. |  |  |  |  |
| Communicate effectively and respectfully with members of the interprofessional health and research team in the conduct of your research. |  |  |  |  |
| Navigate the clinical environment professionally and respectfully. |  |  |  |  |
| Conduct yourself with due regard for ethical practices including patient consent, privacy, and data stewardship. |  |  |  |  |
| Show personal commitment to the conduct of research that is equitable, diverse, and inclusive. |  |  |  |  |

2. Is there anything else you would like to tell us about your readiness for clinical integration?

**CLINICAL RESEARCH SKILLS – MSC1121H**

**READINESS* FOR CLINICAL INTEGRATION POST-COURSE SURVEY**

Integrating into the clinical environment for the first time can be daunting for a new researcher. In this course, we aim to provide you with the basic knowledge, skills and tools necessary to begin your journey in clinical research where you will engage with real patients and their family/support networks, as well as with health care team that cares for them.

**The term ‘readiness’ infers your level of preparedness and how capable you feel. It is not a measure of your enthusiasm or eagerness.*

The following questions aim to evaluate your readiness to engage in the clinical research setting.

1. Please rate your level of readiness for the following:

|  | 1 – not at all ready | 2 – not very ready | 3 - fairly ready | 4 – totally ready |
| --- | --- | --- | --- | --- |
| Interact respectfully with patients and their family/support network in the conduct of your research. |  |  |  |  |
| Communicate effectively with patients and their family/support network in the conduct of your research. |  |  |  |  |
| Communicate effectively and respectfully with members of the interprofessional health and research team in the conduct of your research. |  |  |  |  |
| Navigate the clinical environment professionally and respectfully. |  |  |  |  |
| Conduct yourself with due regard for ethical practices including patient consent, privacy, and data stewardship. |  |  |  |  |
| Show personal commitment to the conduct of research that is equitable, diverse, and inclusive. |  |  |  |  |

2. Is there anything else you would like to tell us about your readiness for clinical integration?

[freetext]

3. Please rate your experience with the VR modules

|  | 1 – not at all | 2 – a little bit | 3 – somewhat helpful | 4 – very helpful |
| --- | --- | --- | --- | --- |
| To what extent did the VR exercises assist with your readiness for clinical integration? |  |  |  |  |

4. Is there anything else you would like to tell us about your experience with the VR exercises?

[freetext]
