# Supplementary material for: Virtual Reality for Developing Patient-facing Communication Skills in a Medical Science Graduate Education Course: A Mixed-Methods Pre-Post Study
Source: Med Sci Educ. 2025 Dec 29;36(1):201–12. doi: 10.1007/s40670-025-02604-4 (PMC13043831; doi:10.1007/s40670-025-02604-4)
Supplement: Supplementary file 4 — Supplementary Material 4. [file 40670_2025_2604_MOESM4_ESM.docx]

Virtual Reality for Developing Patient-Facing Communication Skills in a Medical Science Graduate Education Course: A Mixed-Methods Pre-Post Study

Authors: Kyla Gaeul Lee, Maryam Sorkhou, Nicole Harnett, Sobiga Vyravanathan, Theodore J. Brown, Evan Tannenbaum, Nairy Khodabakhshian*

*Corresponding Author: Nairy Khodabakhshian

Institute of Medical Science, C. David Naylor Building, University of Toronto

6 Queen’s Park Crescent, Suite 119, M5S 3H2, Toronto, Canada

nairy.khodabakhshian@mail.utoronto.ca

**Course Evaluation**

*****Note: responses to all items are on a 5-point likert scale (ranging from: Not at all, Somewhat, Moderately, Mostly, A Great deal), unless indicated otherwise.

Part A

1. I found the course intellectually stimulating
2. The course provided me with a deeper understanding of the subject matter.
3. The instructor A created an atmosphere that was conducive to my learning.
4. The instructor B created an atmosphere that was conducive to my learning.
5. Course projects, assignments, tests, and/or exams improved my understanding of the course material.
6. Course projects, assignments, tests, and/or exams provided opportunity for me to demonstrate an understanding of the course material.
7. Overall, the quality of my learning experience in this course was: Poor, Fair, Good, Very Good, Excellent
8. Please comment on the overall quality of the instruction in this course. [free text]
9. Please comment on any assistance that was available to support your learning in this course. [free text]

Part B

1. The instructor A generated enthusiasm for learning in the course.
2. The instructor B generated enthusiasm for learning in the course.
3. Compared to other courses, the workload for this course was: Very light, Light, Average, Heavy, Very Heavy
4. I would recommend this course to other students: Not at all, Somewhat, Moderately, Mostly, Strongly

Part C

1. The course instructor A made it clear what students were expected to learn in the course.
2. The course instructor B made it clear what students were expected to learn in the course.
3. The course instructor A explained concepts clearly.
4. The course instructor B explained concepts clearly.

Part D

1. The course instructor A organized lectures in a logical manner.
2. The course instructor B organized lectures in a logical manner.
3. The course instructor A expressed interest in students’ learning approaches (e.g., note-taking, study habits) throughout the course.
4. The course instructor B expressed interest in students’ learning approaches (e.g., note-taking, study habits) throughout the course.
5. The course instructor A related course concepts to current issues or real-life situations.
6. The course instructor B related course concepts to current issues or real-life situations.
