# Supplementary material for: Virtual Reality for Developing Patient-facing Communication Skills in a Medical Science Graduate Education Course: A Mixed-Methods Pre-Post Study
Source: Med Sci Educ. 2025 Dec 29;36(1):201–12. doi: 10.1007/s40670-025-02604-4 (PMC13043831; doi:10.1007/s40670-025-02604-4)
Supplement: Supplementary file 5 — Supplementary Material 5. [file 40670_2025_2604_MOESM5_ESM.docx]

Dear Ms. Khodabakhshian,

We have received the reports from our advisors on your manuscript, "Virtual Reality for Developing Patient-Facing Communication Skills in a Medical Science Graduate Education Course: A Mixed-Methods Pre-Post Study", which you submitted to Medical Science Educator.

Based on the advice received, I have decided that your manuscript could be reconsidered for publication should you be prepared to incorporate major revisions. When preparing your revised manuscript, you are asked to carefully consider the reviewer comments which can be found below, and submit a list of responses to the comments. You are kindly requested to also check the website for possible reviewer attachment(s).Please note: When uploading your revised manuscript files, please make sure only to submit your editable source files (i.e. Word, Tex). PDF is not allowed at this stage.

In order to submit your revised manuscript, please access the following web site link below.

Your username is: nairy.khodabakhshian

If you forgot your password, you can click the 'Send Login Details' link on the EM Login page at [https://www.editorialmanager.com/mdse/](https://can01.safelinks.protection.outlook.com/?url=https%3A%2F%2Fwww.editorialmanager.com%2Fmdse%2F&data=05%7C02%7Ckyla.lee%40mail.utoronto.ca%7C1f9469bf88c64f273d0708de0a709c3a%7C78aac2262f034b4d9037b46d56c55210%7C0%7C0%7C638959675944258343%7CUnknown%7CTWFpbGZsb3d8eyJFbXB0eU1hcGkiOnRydWUsIlYiOiIwLjAuMDAwMCIsIlAiOiJXaW4zMiIsIkFOIjoiTWFpbCIsIldUIjoyfQ%3D%3D%7C0%7C%7C%7C&sdata=k0CXq0%2BqnkqpQT3b0NROcRDq4r7CZLhZuSOlyoYTacE%3D&reserved=0).

We look forward to receiving your revised manuscript before 27 Nov 2025.

With kind regards,

Machelle Linsenmeyer, Ed.D, FNAOME

Associate Editor

Dear Dr. Linsenmeyer,

Thank you for your careful review of this paper. We have responded to comments in this letter, in blue. We have made revisions using tracked changes for your and other reviewers’ conveniences and are also submitting a clean version.

COMMENTS FOR THE AUTHOR:

***Reviewer #2:***

Reviewer #2: Lee et al. investigated the application of virtual reality (VR) as a technique for enhancing communication skills among patients, proposing its integration into medical education for graduate students. As AI-driven tools and educational modules gain traction within healthcare training, this manuscript provides pertinent insights into the topic. While the overall concept is not groundbreaking, the development of these modules serves as an additional example of AI utilization in medical education. However, the manuscript suffers from considerable flaws, both in clarity and structure.

1. The study design is inadequately described, particularly concerning the two VR modules that form the foundational aspect of the research. There is a lack of detail regarding the rationale behind the construction of each patient scenario, including the specific objectives they aim to address.

- Thank you for this feedback. We have revised the Study Design section to include more detail regarding the rationale behind the construction of each patient scenario and contents of each Module, and have also included the specific educational objectives the Modules aimed to address. We have also added a figure to illustrate the decision points and outcomes for both Modules.

2. The introductory paragraph requires significant revision or removal due to its lack of clarity, particularly in differentiating the roles of clinical practitioners versus researchers in healthcare environments. A more thorough elucidation of their respective responsibilities, daily tasks, and the outcomes tied to each role would enhance understanding. This clarification is crucial, especially as the authors advocate for a VR module that emulates clinical experiences.

- The introduction has been revised to improve clarity and focus on clinical research. The differences between the roles of clinical practitioners versus clinical researchers is discussed.

3. Moreover, the manuscript contains numerous instances of missing citations. For instance, the following statements require appropriate references:

- "Despite the recognized importance of these communication skills, they are often inadequately developed through traditional teaching methods."

- "Such methods often lack the capacity to simulate the complexities of actual clinical interactions, leaving a gap in students' preparedness for professional practice."

- "Further, incidental observation is often difficult to regulate and rarely the same between two students, resulting in inconsistent learning experiences."

- "There are also often administrative barriers that delay the learning process and place additional strain on healthcare workers tasked with training graduate students."

- "Virtual reality (VR) has emerged as a transformative technology in medical education, offering an interactive, experiential, and immersive learning environment that can address these educational gaps."

- These sentences have either been removed or cited accordingly.

4. Additionally, the section detailing the study design needs further elaboration beyond a single sentence. It should briefly outline the target cohort, the nature of comparisons made, and the aims of the research.

- We thank the reviewer for bringing up this important point. The *Study Design* section has now been expanded to include a clearer depiction of the target cohort, the nature of comparisons made, and the aims of the study. The revised text now reads as follows:

““A mixed methods pre-post study design was used to evaluate the effectiveness of the MSC1121H *Clinical Research Skills* course in strengthening graduate students’ communication skills for patient-facing research. Research ethics approval was obtained from the University of Toronto Research Ethics Board (Protocol #: 00047058). The study examined first- and second-year graduate students who had completed the course over two terms. Quantitative measures captured changes in students’ knowledge and readiness for clinical integration. Qualitative data from class debrief sessions explored students’ experiences in navigating the virtual reality modules, their perceptions of ethical and cultural challenges, and reflections on how these scenarios shaped their understanding of patient communication in clinical research settings. These two approaches aimed to elucidate whether and how virtual reality–based learning influences skill development and attitudes in patient-facing communication.”

5. It also remains unclear if participants were provided with a consent form for their involvement in the VR course, which is a critical ethical consideration.

- Thank you for raising this very important point. Participant consent was implied through enrollment in the course and completion of course activities, as the data collection activities were embedded within the regular course curriculum and followed the course schedule. Prior to data collection, students were informed about the purpose of data collection (i.e., for course improvement, evaluation, and research) and were given the option to opt out of having their data included in the research analysis. We have revised to include this in the Methods, under Participants.

6. Further information is necessary regarding the inclusion of patients and caregivers in both VR modules. The manuscript lacks explanation about how and why each element of the patient scenario was constructed. For example, the purpose of portraying the patient's caregiver as skeptical and the inclusion of an ethical dilemma need to be justified.

- Thank you for this comment. We have added additional details to the VR modules to describe our reasoning for the elements of each patient scenario, including why oncology was chosen as a research setting for module 1, why a granddaughter was included in module 2, why the granddaughter was skeptical, and the inclusion of the ethical dilemma in module 2. We have also revised to include the specific objectives of each module.

7. Finally, if the study compares pre- and post-module experiences, the methodology for assessing the efficacy of the VR module needs to be clearly defined. It is important to consider whether pre- and post-module didactic lectures or readings might yield comparable or improved outcomes, thus questioning the unique impact of the VR intervention.

- We thank the reviewer for this valuable comment. We have clarified in the Data Collection: Quantitative Measures and Data Analysis section the sequencing of lectures, discussions, and VR activities, along with an explanation of how the pre-course and post-course measures reflect baseline and cumulative learning, respectively.

To further acknowledge this point, we have expanded on one of our statements in the Study Limitations section noting that the assessments were not conducted immediately before and after the VR modules. As a result, this limits our ability to disentangle the unique effects of the VR component. The revised limitation reads:

“Third, because the pre- and post-course assessments captured cumulative learning from the entire course rather than changes immediately before and after the VR modules, it was not possible to disentangle the unique impact of the VR modules to observed improvements. The pre-course surveys were administered at the start of the semester prior to any lectures or discussions, while the VR activities occurred near the end of the course. As a result, the gains that occurred post-course likely reflect the combined influence of both didactic instruction and experiential learning from completion of the VR modules.”

We hope that these revisions clarify the methodological framework and explicitly address the limitations of attributing changes solely to the VR intervention.

**Reviewer #3:**

Reviewer #3: For Research Articles: Feedback on the design, methods and statistics:

This manuscript would be significantly improved by a more detailed explanation of the setup of the VR modules. How exactly are students interacting with the VR system? Are there pre-determined responses that they have to choose between? Are they able to give free responses? The mechanics of the interaction could significantly impact learning outcomes.

Were there any changes made to the course or modules between the 1st and 2nd offering?

- The “VR Modules” section in the “Methods” has been revised to include answers to these questions.

Feedback on the results/discussion or main body of the manuscript:

The module success rates are concerning, and the authors do not address why these success rates are so low and what this means for module design.

- Thank you for bringing this up. We have revised the discussion section to address reasons why the success rates may be low, and what this means for the course design for both Module 1 and 2.

It would be beneficial to discuss cost-effectiveness and scalability of this project. What are the resource requirements compared to the traditional teaching methods.

- Thank you for bringing up this very interesting and important point. We have added a section in the discussion and future directions about the potential cost-effectiveness and scalability of VR compared to other traditional teaching methods.

The lengthy discussion of cancer disclosure practices seems out of place in light of the remainder of the manuscript.

- Thank you for your feedback. We agree, and we have revised the discussion to recenter the findings of our study as it relates to Module 2, as opposed to discussing cancer disclosure practices in healthcare.

What are some ways for addressing the cultural safety knowledge gap identified in the study?

- Thank you for raising this question. We have revised the discussion to discuss ways for addressing the cultural safety knowledge gap identified in our study.

***Reviewer #4:***

Reviewer #4: Feedback on the introduction/background/rationale/theoretical framework: The introduction is thorough and well-structured. It clearly establishes the importance of communication skills in medical science graduate education and highlights the limitations of traditional teaching methods. The rationale for using virtual reality (VR) as an innovative solution is compelling, supported by references to gaps in experiential learning and the need for culturally competent communication. Overall, the background provides a strong justification for the study and situates it well within current educational challenges.

For Research Articles: Feedback on the design, methods and statistics: The study employs a mixed-methods pre-post design, which is appropriate for evaluating both quantitative and qualitative outcomes. The description of the course, participant selection, and VR modules is detailed and transparent. Quantitative measures (knowledge test and readiness survey) are clearly described, and the use of paired sample t-tests is suitable for pre-post comparisons. The qualitative component—class debrief and thematic analysis—is well-explained, with field notes coded inductively. Limitations are acknowledged, including the absence of a control group, small sample size, and reliance on self-reported data. The statistical analysis is sound, but the manuscript could be strengthened by including more information on the validity and reliability of the instruments used, as well as a discussion of potential confounders.

- We thank the reviewer for raising this important point. The Knowledge test was developed by the course team to reflect course-specific content and therefore was not subjected to formal psychometric validation. Similarly, the Readiness for Clinical Integration Survey was adapted from an internal evaluation tool used in the University of Toronto’s Temerty Faculty of Medicine’s MD program. However, this measure has not been formally validated or reliability-tested in prior research. Nonetheless, internal consistency was examined in the present study and was found to be acceptable (Cronbach’s α = 0.70 pre-course; α = 0.75 post-course). These analyses and findings are reported in “Data Collection: Quantitative Measures and Data Analysis” and “Quantitative Findings,” respectively.

Concerning potential confounds, we have provided a more thorough description of variables that may have influenced our findings within the limitation section of the manuscript. The limitation section now includes the following: “Fourth, since data were collected across two academic terms, cohort differences and timing effects may have influenced outcomes. Fifth, our findings may have been confounded by variation in students’ prior research experience. Although the course primarily enrolled first- and second-year graduate students, it is possible that some students had previous professional or academic experience, which may have influenced their baseline readiness and learning gains.”

The integration of qualitative and quantitative findings is a notable strength.

Feedback on the results/discussion or main body of the manuscript: Results are presented clearly, with significant improvements in both knowledge and readiness scores post-intervention. The discussion effectively interprets these findings, linking them to the broader literature on VR in medical education. The qualitative results provide rich insights into students' experiences, challenges with cultural safety, and role confusion between researcher and clinician. The discussion is reflective and acknowledges areas for improvement, such as the need for more comprehensive training in patient-centered communication and ethical consent. The manuscript thoughtfully addresses the implications of excluding certain populations and the importance of equity, diversity, and inclusion. The integration of student reflections adds depth to the analysis. However, the discussion could be enhanced by further exploring the long-term impact of VR training and its scalability.

- Thank you for this note. We have revised the discussion to further explore the long-term impact of VR training and scalability by discussing its potential for cost-effectiveness.

Feedback on the conclusions/summary: The conclusions are well-aligned with the study's findings. The manuscript emphasizes VR's potential as a transformative tool for teaching non-technical skills, such as communication, ethical decision-making, and cultural safety. Limitations are candidly discussed, and future directions are proposed, including the need for controlled studies and exploration of long-term outcomes. The summary is concise and appropriately highlights the significance of the research for medical science education. It also suggests practical applications and areas for further investigation.

Feedback on the appropriateness and timeliness of the reference list: The reference list is comprehensive and current, including recent studies on VR in education, communication skills, and cultural safety. The citations cover both foundational and contemporary literature, supporting the manuscript's arguments and situating the study within ongoing scholarly discourse. References to international and culturally diverse contexts are particularly relevant given the study's focus on cultural safety. The inclusion of government guidelines and policy statements adds credibility. Overall, the references are appropriate and timely.

Feedback on the tables and figures: Tables and figures are used effectively to present quantitative results (e.g., pre- and post-course scores for knowledge and readiness). Figures are clear, with appropriate labeling and error bars. The visual presentation aids in understanding the statistical findings. However, the manuscript could benefit from additional tables summarizing qualitative themes or student feedback for greater clarity in a tabular format.

- Thank you for this suggestion. We have added a table that summarizes the qualitative themes that emerged.

Summary: This manuscript presents a well-designed and timely study on the use of VR for developing patient-facing communication skills in medical science graduate education. The strengths include a clear rationale, robust mixed-methods design, thoughtful discussion, and relevant references. Areas for improvement include further detail on instrument validity, exploring the long-term impact of VR training and its scalability, and enhanced integration of qualitative findings in tabular form. Overall, the manuscript makes a valuable contribution to the field and is suitable for publication with minor revisions.

Associate Editor: While all three reviewers have clearly outlined points to help improve the manuscript, please ensure that particular attention is paid to reviewer 2 and 3 concerns and ensure that those are carefully considered and addressed.
